# *Dalbergia odorifera* Volatile Oil Alleviates Microsphere-Induced Myocardial Microcirculatory Dysfunction via Inhibiting Neutrophil Extracellular Traps Formation

**DOI:** 10.3390/ph19060959

**Published:** 2026-06-20

**Authors:** Yinju Liu, Wei Hou, Zengcai Liu, Yanlong Zhou, Xing Dai, Dongdong Jia, Lanying Chen, Ronghua Liu

**Affiliations:** 1 School of Pharmacy, Jiangxi University of Chinese Medicine, Nanchang 330004, China; liuyinju@jxutcm.edu.cn (Y.L.); daixing1@jxutcm.edu.cn (X.D.); 2 National Pharmaceutical Engineering Center for Solid Preparation of Chinese Herbal Medicine, Jiangxi University of Traditional Chinese Medicine, Nanchang 330006, China; houwei@jxutcm.edu.cn (W.H.); liucengcai@jxutcm.edu.cn (Z.L.); zhouyanlong@jxutcm.edu.cn (Y.Z.); jiadongdong@jxutcm.edu.cn (D.J.); 3 Jiangxi Provincial Key Laboratory of Effective Material Basis of TCM (2024SSY07102), Jiangxi University of Chinese Medicine, Nanchang 330004, China

**Keywords:** *Dalbergia odorifera* volatile oil, myocardial microcirculatory dysfunction, neutrophil extracellular traps (NETs), improvement

## Abstract

**Background/Objectives**: Myocardial microcirculatory dysfunction is a critical pathological feature of cardiovascular diseases, closely associated with inflammation, oxidative stress, and excessive neutrophil activation. Neutrophil extracellular traps (NETs) serve as crucial mediators of myocardial microvascular inflammatory injury. *Dalbergia odorifera* volatile oil (DOVO) demonstrates anti-inflammatory and antioxidant properties; however, its protective role against myocardial microcirculatory damage and its regulatory effect on NET formation remain inadequately characterized. This study investigates the protective effects of DOVO on myocardial microcirculatory disturbances and elucidates the underlying mechanisms related to NETs. **Methods**: A rat model of myocardial microcirculatory dysfunction was established through polyethylene microsphere injection, and an in vitro neutrophil inflammation model was generated using differentiated HL-60 cells. DOVO was administered at various doses both in vivo and in vitro, and hemodynamics, inflammatory cytokines, oxidative stress, and NET-related markers, including MPO and CitH3, were analyzed. **Results**: DOVO dose-dependently ameliorated microcirculatory impairment, hemodynamic disorders, inflammation, and oxidative stress in rats, significantly suppressing NET formation. In differentiated HL-60 cells, DOVO similarly reduced inflammatory gene expression and inhibited LPS-induced NETs production by downregulating MPO and CitH3. **Conclusions**: DOVO suggests a protective effect against myocardial microcirculatory injury by inhibiting oxidative stress, inflammatory responses, and subsequent NET formation. These findings elucidate a novel mechanism by which DOVO alleviates microcirculation-related cardiac damage and provide a theoretical basis for its application in cardiovascular injury.

## 1. Introduction

Microcirculatory dysfunction, characterized by abnormalities in the perfusion, structure, and function of microvessels, constitutes a fundamental pathological state that disrupts the exchange of substances between blood and tissue cells, leading to cumulative damage across multiple organs and systems [1]. Notably, coronary microcirculatory dysfunction, a specific subtype targeting the microvascular network of the myocardium, is marked by impaired coronary blood flow regulation, abnormal endothelial function, and reduced microvascular perfusion, which directly compromises myocardial oxygen supply and metabolic homeostasis. As a crucial driver of the onset and progression of various chronic disorders—such as cardiovascular and cerebrovascular diseases, as well as diabetic microvascular complications—microcirculatory dysfunction, including its coronary subtype, initially manifests as cellular ischemia, hypoxia, and metabolic disturbances. In the context of coronary microcirculatory dysfunction, this early stage is often associated with myocardial microinfarction, angina pectoris, or subtle myocardial dysfunction that may not be detected by conventional coronary angiography. As the condition advances, it inflicts pathological damage on microcirculation-rich organs, including the heart, brain, kidneys, and eyes, potentially triggering severe complications such as cerebral infarction, myocardial ischemia, diabetic foot, and retinopathy; for coronary microcirculatory dysfunction specifically, progression can lead to fulminant myocardial ischemia, heart failure, or even sudden cardiac death [2]. Furthermore, as a key pathological basis for chronic diseases, microcirculatory dysfunction can exacerbate the primary illness, induce microthrombosis, and provoke systemic inflammatory responses—coronary microcirculatory dysfunction, in particular, can aggravate underlying coronary artery disease, increase the risk of recurrent cardiac events. In severe cases, it may lead to systemic hypoperfusion or even shock. Ultimately, this dysfunction establishes a progressive cascade of ‘local injury–systemic dysfunction’, which serves as a significant underlying cause for the recurrence and treatment refractoriness of numerous chronic diseases, including those affecting the coronary circulation.

Neutrophils, as key effector cells of innate immunity, play a crucial role in the initiation and progression of microcirculatory dysfunction [3]. The abnormal activation of neutrophils under pathological conditions constitutes a central event driving microcirculatory disturbances, with significant pathological triggers including inflammatory cytokines, hypoxia, and damage-associated molecular patterns (DAMPs) [4]. Activated neutrophils initially accumulate through a cascade of rolling and firm adhesion to vascular endothelial cells, which directly obstructs microvessels comparable to their own diameter [5,6]. Concurrently, they release a multitude of cytotoxic and pro-inflammatory molecules, such as neutrophil elastase (NE), myeloperoxidase (MPO), and pro-inflammatory cytokines [7], which directly induce oxidative damage to endothelial cells, degrade endothelial junction proteins [8] and basement membrane structures [9,10,11], and compromise microcirculatory integrity. This process amplifies the inflammatory cascade and exacerbates the microenvironment [6]. Notably, Neutrophil Extracellular Traps (NETs), which are web-like structures composed of chromatin DNA decorated with histones and granule proteins, inflict multifaceted damage by occluding microvessels [12,13], activating endothelial coagulation pathways [12,14], and promoting microthrombosis [6], while also directly impairing the vascular endothelium through histones and proteases. Additionally, NETs recruit and further activate immune cells by acting as damage-associated molecular patterns [6]. Targeting neutrophil activation, adhesion, degranulation, and particularly the formation and clearance of NETs may represent a promising exploratory strategy for alleviating microcirculatory dysfunction, providing a valuable theoretical foundation and potential therapeutic target for novel interventions, including those derived from Traditional Chinese Medicine (TCM).

*Dalbergia odorifera* T. Chen (Fabaceae) is a medicinal plant whose dried heartwood from stems and roots is widely utilized in traditional medicine [15]. Modern pharmacological studies have demonstrated that this plant is rich in volatile oils, flavonoids, terpenoids, and other bioactive constituents [16,17,18], which endow it with diverse biological activities, including vasorelaxant [19], antithrombotic [20], antioxidant [21], anti-inflammatory [22,23,24], and anti-tumor [25] effects. As the primary active fraction, the essential oil of *D. odorifera*, mainly composed of volatile sesquiterpenoids and aromatic compounds, exhibits significant anti-oxidative stress, anti-inflammatory, and vascular endothelial protective effects, which are regarded as the key material basis for its pharmacological activities [18]. It is important to note that while the traditional and clinical uses of *D. odorifera* primarily refer to the crude herbal material decoction or its extracts for promoting blood circulation and relieving pain, its volatile oil (DOVO) is considered a key active fraction responsible for many of these pharmacological effects. Currently, DOVO has been incorporated into several representative preparations for the clinical adjunctive treatment of microcirculation-related diseases [26,27]. Although *D. odorifera* volatile oil has been widely utilized in various classic medicinal formulations and clinical practices for managing cardiovascular injury, its underlying molecular mechanisms remain complex and poorly characterized. The lack of sufficient evidence regarding its specific molecular targets and signaling pathways in regulating microcirculatory dysfunction has impeded a deeper mechanistic understanding and the rational development of this promising agent. This study focuses on the core target of Neutrophil Extracellular Traps (NETs), investigating the inhibitory effects of *D. odorifera* volatile oil on NET formation and its associated damaging consequences. The aim is to elucidate the potential molecular mechanisms underlying the “Pungent-Aromatic Unblocking of Collaterals (Xinxiang Tongluo)” property of this volatile oil. This research aspires to provide new therapeutic targets and insights for Traditional Chinese Medicine (TCM)-based interventions against microcirculatory dysfunction, while also offering experimental support for the rational clinical application and novel drug development involving *D. odorifera* volatile oil.

## 2. Results

### 2.1. HPLC Chemical Composition Analysis of D. odorifera Volatile Oil

**Figure 1:**
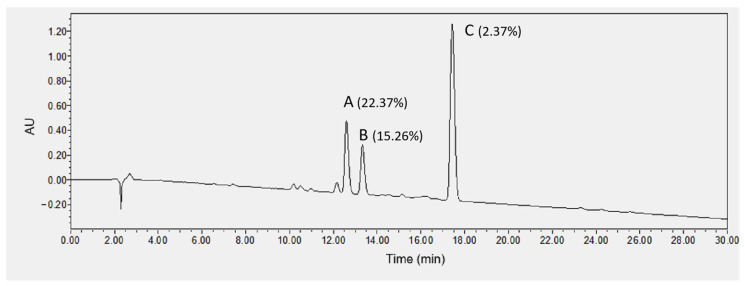
Chromatographic analysis of volatile components in the vascular organoid culture system. The y-axis represents absorbance units (AUs), and the x-axis represents retention time (min). Three major chromatographic peaks (labeled A, B, C) are indicated, corresponding to the following compounds: Peak A: (3S,6R,7R)-3,7,11-trimethyl-3,6-epoxy-1,10-dodecadien-7-ol; Peak B: (3S,6S,7R)-3,7,11-trimethyl-3,6-epoxy-1,10-dodecadien-7-ol; Peak C: trans-nerolidol.

The chemical composition of *D. odorifera* volatile oil (DOVO) was analyzed by high-performance liquid chromatography (HPLC). As shown in Figure 1, three major chromatographic peaks were detected at a wavelength of 210 nm. By comparison of retention times and UV spectra with reference standards, Peak A was identified as (3S,6R,7R)-3,7,11-trimethyl-3,6-epoxy-1,10-dodecadien-7-ol, Peak B as (3S,6S,7R)-3,7,11-trimethyl-3,6-epoxy-1,10-dodecadien-7-ol, and Peak C as trans-nerolidol. Based on peak area normalization, the relative contents of the three compounds were approximately 22.37%, 15.26%, and 62.37%, respectively. These are the percentages concerning the sesquiterpenes in DOVO. At the fixed detection wavelength of 210 nm, these three identified sesquiterpenoids represented 91% of the total peak area, though other compounds that are not detectable at this wavelength may also be present in DOVO.

### 2.2. Ameliorative Effects of D. odorifera Volatile Oil on Microcirculatory Disturbance and Hemodynamic Disorders in Rats

**Figure 2:**
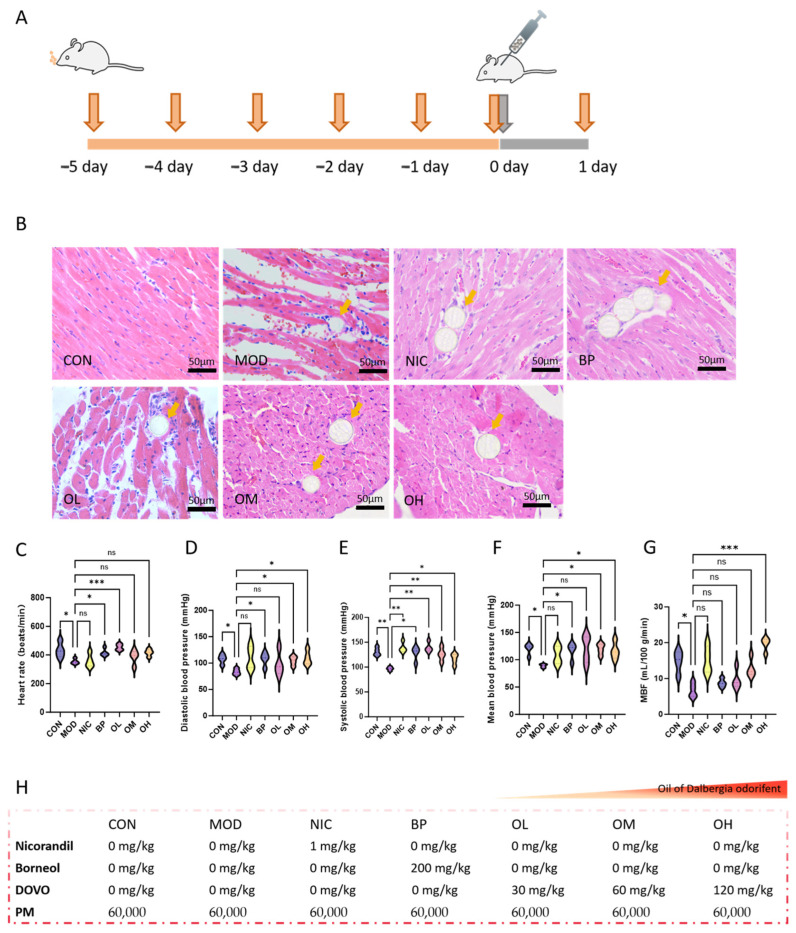
Effects of *D. odorifera* volatile oil (DOVO) on myocardial microcirculatory disturbance and hemodynamic disorders in rats. (**A**) Timeline of the animal experimental protocol; (**B**) Representative hematoxylin–eosin (HE) staining of myocardial tissue sections (scale bar = 50 μm, yellow arrows indicate the polyethylene microspheres); (**C**) Heart rate (* *p* < 0.05, *** *p* < 0.001, N = 4); (**D**) Diastolic blood pressure (* *p* < 0.05, N = 4); (**E**) Systolic blood pressure (* *p* < 0.05, ** *p* < 0.01, N = 4); (**F**) Mean arterial pressure (* *p* < 0.05, N = 4); (**G**) Myocardial blood flow (MBF) (* *p* < 0.05, *** *p* < 0.001, N = 4); (**H**) Overview of group assignments, drug doses, and modeling conditions. (CON, control; MOD, model; NIC, nicorandil; BP, borneol; OL/OM/OH, low-/medium-/high-dose DOVO. Data are presented as violin plots; ns, not significant).

Rats were administered oral water (control group without microspheres, MOD), *D. odorifera* volatile oil (DOVO) at low (OL), medium (OM), and high (OH) doses, Nicorandil (NIC), and Borneol (Bingpian, BP). On the sixth day, 45 min after oral administration, polyethylene microspheres (PMs) were injected into the heart to induce microcirculatory disorders in the rats. Administration continued for one additional day to observe the effects of *D. odorifera* volatile oil on microcirculatory disorders (Figure 2A). Representative hematoxylin–eosin (HE) staining of myocardial tissue sections from each experimental group is presented in Figure 2B. The CON group exhibited normal myocardial tissue morphology, with neatly arranged myocardial fibers, clear cell boundaries, no polyethylene microspheres, and no obvious inflammatory cell infiltration or structural damage. In contrast, the MOD group showed severe pathological changes around the injected polyethylene microspheres, including disordered myocardial fiber arrangement, myocardial fiber swelling and rupture, extensive red blood cell exudation and hemorrhage, and significant inflammatory cell infiltration, indicating successful establishment of the myocardial microcirculatory disorder model. Compared with the MOD group, the NIC (nicorandil) and BP (borneol) positive control groups, as well as the OL (low-dose DOVO), OM (medium-dose DOVO), and OH (high-dose DOVO) treatment groups, all showed dose-dependent improvements in pathological damage around the polyethylene microspheres. Specifically, the OL group still presented mild myocardial fiber disorder and inflammatory cell infiltration around the microspheres, while the OM group exhibited reduced hemorrhage and inflammatory infiltration with more orderly myocardial fibers near the microspheres. The OH group demonstrated the most remarkable therapeutic effect, with myocardial fiber arrangement around the microspheres approaching that of the CON group, minimal red blood cell exudation, and almost no obvious inflammatory cell infiltration, indicating that high-dose *D. odorifera* volatile oil effectively alleviated myocardial microcirculatory disorder-induced pathological injury around the polyethylene microspheres (Figure 2B). Hemodynamic assessments further demonstrated that, compared to the control group, the MOD group exhibited significant decreases in heart rate (Figure 2C), diastolic blood pressure (Figure 2D), systolic blood pressure (Figure 2F), and mean arterial pressure (Figure 2E) (all *p* < 0.05 or *p* < 0.01). However, pretreatment with *D. odorifera* volatile oil modulated these parameters in a dose-dependent manner: heart rate showed recovery trends in the BP, OL, OM, and OH groups (with the OH group demonstrating the most notable increase, ** *p* < 0.001 vs. MOD) (Figure 2B); diastolic blood pressure was significantly improved in the BP and OL groups (*p* < 0.05 vs. MOD) (Figure 2C); systolic blood pressure was dose-dependently reversed in the NIC, BP, OL, OM, and OH groups (with OM and OH groups showing more significant improvements, * *p* < 0.01 vs. MOD) (Figure 2D); and mean arterial pressure was elevated in the BP, OL, OM, and OH groups, with the OH group achieving the most pronounced recovery (*p* < 0.05 vs. MOD) (Figure 2E). Furthermore, the myocardial microvascular blood flow was significantly reduced in the MOD group compared with the CON group (*p* < 0.001). Treatment with NIC, BP, and DOVO (OL, OM, OH) dose-dependently restored the blood flow, with the OH group showing the most pronounced improvement (*p* < 0.01 vs. MOD, Figure 2G). These results suggest that DOVO may improve microcirculatory perfusion.

### 2.3. Inhibitory Effects of D. odorifera Volatile Oil on Neutrophil Recruitment and Inflammatory Responses in Rats

**Figure 3:**
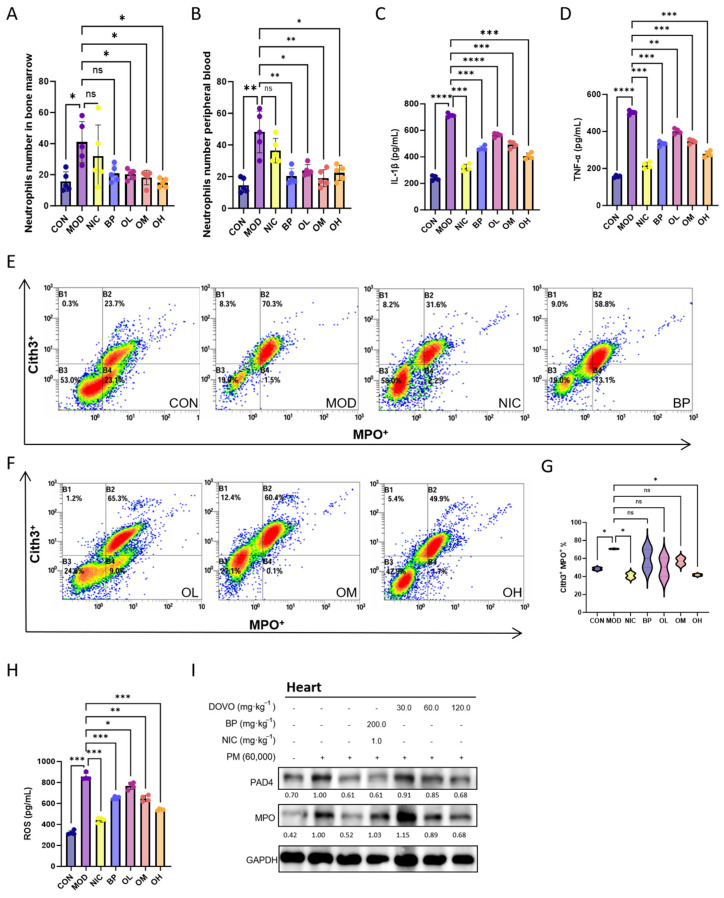
Effects of *D. odorifera* volatile oil (DOVO) on neutrophil recruitment, inflammation, and neutrophil extracellular trap (NET) formation in vivo. (**A**) Neutrophil counts in bone marrow (* *p* < 0.05, N = 4); (**B**) Neutrophil counts in peripheral blood (* *p* < 0.05, ** *p* < 0.01, N = 4); (**C**) Serum interleukin-1β (IL-1β) levels (*** *p* < 0.001, **** *p* < 0.0001, N = 4); (**D**) Serum tumor necrosis factor-α (TNF-α) levels (** *p* < 0.01, *** *p* < 0.001, **** *p* < 0.0001, N = 4); (**E**,**F**) Representative flow cytometry plots showing CitH3^+^MPO^+^ double-positive neutrophils in peripheral blood; (**G**) Quantification of CitH3^+^MPO^+^ double-positive cell proportion (* *p* < 0.05, N = 4); (**H**) Serum reactive oxygen species (ROS) levels (* *p* < 0.05, ** *p* < 0.01, *** *p* < 0.001, N = 4); (**I**) Western blot analysis of PAD4 and MPO protein expression in myocardial tissues, with GAPDH as the internal reference. (CON, control; MOD, model; NIC, nicorandil; BP, borneol; OL/OM/OH, low-/medium-/high-dose DOVO. Data are presented as mean ± SEM or violin plots; ns, not significant).

In comparison to the normal COM group, the MOD group exhibited significantly elevated neutrophil counts in both bone marrow and peripheral blood (Figure 3A,B), along with increased serum levels of interleukin-1β (IL-1β) (Figure 3C) and tumor necrosis factor-α (TNF-α) (* *p* < 0.05 or ** *p* < 0.001) (Figure 3D). In contrast to the MOD group, pretreatment with NIC, BP, OL, OM, and OH groups resulted in a dose-dependent reduction in neutrophil counts in both bone marrow and peripheral blood (Figure 3A,B), as well as downregulation of serum levels of IL-1β (Figure 3C) and TNF-α (Figure 3D). Flow cytometric analysis revealed that, compared to the normal CON group, the proportion of double-positive CitH3^+^MPO^+^ neutrophils in the peripheral blood of rats in the MOD group was significantly elevated (* *p* < 0.05) (Figure 3E,G), indicating that polyethylene microsphere-induced microcirculatory disorders may trigger excessive formation of NETs. In comparison to the MOD group, pretreatment with NIC, BP (Figure 3F,G), OL, OM, and OH (Figure 3F,G) all significantly reduced the proportion of CitH3^+^MPO^+^ double-positive cells, with the OM and OH groups demonstrating more pronounced inhibitory effects. Furthermore, serum reactive oxygen species (ROS) detection revealed that ROS levels in the MOD group were significantly higher compared to the CON group (** *p* < 0.001), Notably, NIC, BP, and all DOVO dose groups were able to downregulate serum ROS content in a dose-dependent manner, suggesting that DOVO may mitigate the upstream triggers of NETs generation by inhibiting oxidative stress (Figure 3H). Western blot analysis demonstrated that the expression of peptidylarginine deiminase 4 (PAD4), a key regulatory enzyme for NET formation, and myeloperoxidase (MPO), a core functional component of NETs, was significantly upregulated in the cardiac tissues of the MOD group. In comparison to the MOD group, pretreatment with NIC, BP, and all doses of DOVO resulted in a dose-dependent decrease in the protein expression levels of PAD4 and MPO in cardiac tissues (Figure 3I). Notably, the OH group exhibited the most pronounced inhibitory effects on PAD4 and MPO, with its regulatory efficacy comparable to that of the positive control drug NIC. These findings suggest that DOVO can mitigate the formation and activation of NETs in cardiac tissues by inhibiting the PAD4-MPO pathway, thereby alleviating pathological damage associated with microcirculatory disorders.

### 2.4. Establishment of a Neutrophil-like Differentiation Model from HL-60 Cells

**Figure 4:**
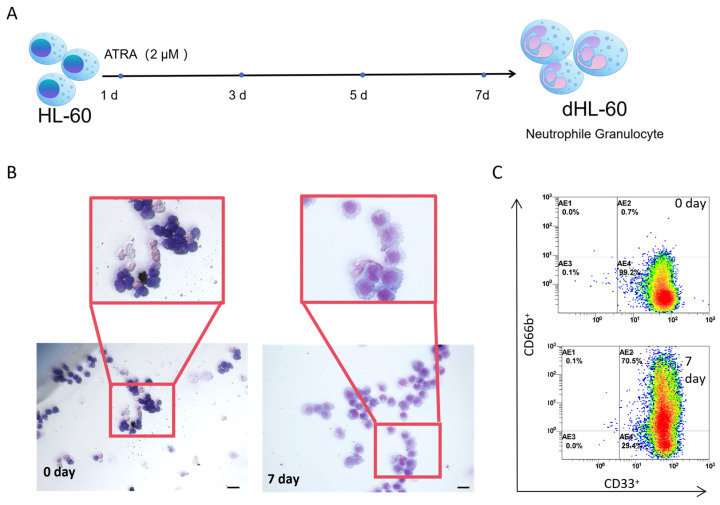
Establishment and identification of the HL-60 neutrophil-like differentiation model. (**A**) Timeline of HL-60 cell differentiation into neutrophil-like cells induced by 2 μM all-trans-retinoic acid (ATRA) for 7 days; (**B**) Wright–Giemsa staining of HL-60 cells at day 0 and day 7 (the red box is the magnified image, bar = 20 μm); (**C**) Flow cytometric analysis of CD33^+^CD66b^+^ double-positive cells in HL-60 cells before (day 0) and after (day 7) ATRA induction, confirming successful differentiation into neutrophil-like dHL-60 cells.

To establish an in vitro model of neutrophil differentiation, HL-60 cells were continuously treated with 2 μM all-trans-retinoic acid (ATRA) for 7 days (Figure 4A), and the differentiation efficiency was evaluated through morphological observation and flow cytometry. The results of Wright–Giemsa staining (Figure 4B) indicated that untreated HL-60 cells (day 0) displayed a round shape with large, deeply stained nuclei and a lack of obvious cytoplasmic granules, which is characteristic of undifferentiated myeloid cells. Following 7 days of ATRA induction, the cells exhibited an increase in volume, with distinct cytoplasmic granules and lobulated nuclei, consistent with the morphological features of mature neutrophils. This observation suggests that ATRA effectively induces the differentiation of HL-60 cells into neutrophils. Flow cytometric analysis of the expression of the myeloid differentiation marker CD33 and the neutrophil-specific marker CD66b (Figure 4C) further corroborated this finding: on day 0, the proportion of CD33^+^CD66b^+^ double-positive cells was only 0.7%, with the majority of cells being CD33 single-positive and remaining in an undifferentiated state. After 7 days of ATRA induction, the proportion of CD33^+^CD66b^+^ double-positive cells significantly increased to 70.5%, demonstrating that HL-60 cells successfully differentiated into neutrophil-like cells (dHL-60). This differentiation model can be utilized for subsequent studies on neutrophil-related functions.

### 2.5. D. odorifera Volatile Oil Inhibits LPS-Induced Inflammation and NET Formation in Differentiated HL-60 Cells

**Figure 5:**
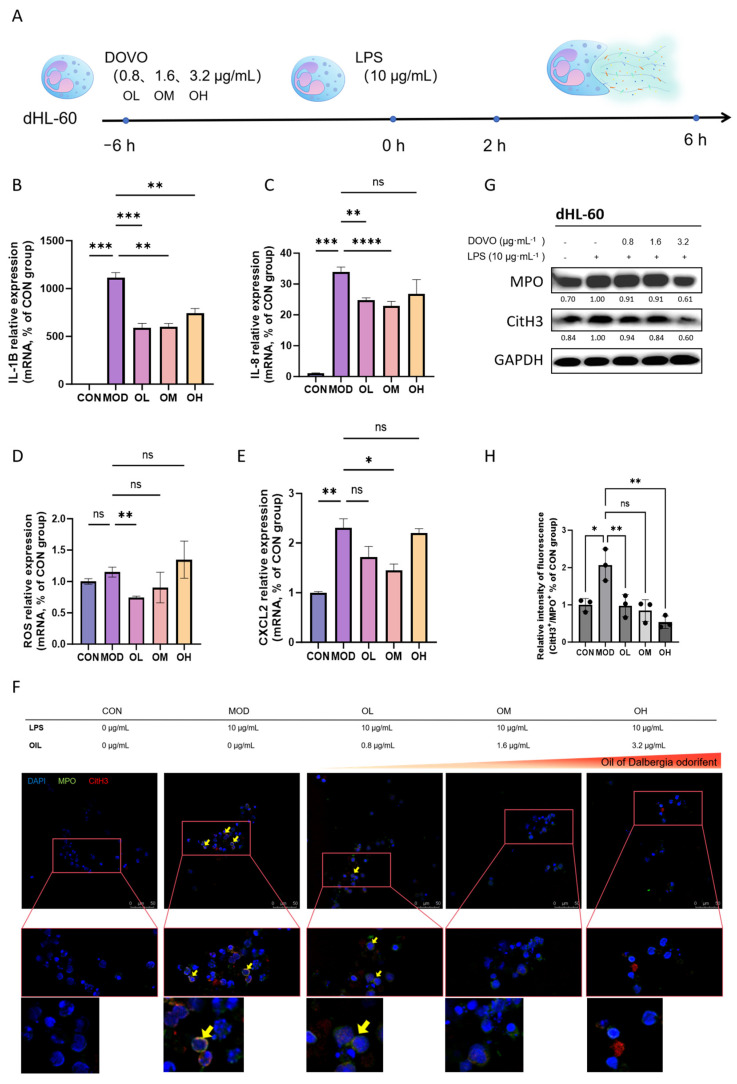
Effects of *D. odorifera* volatile oil (DOVO) on LPS-induced inflammation and NET formation in differentiated HL-60 (dHL-60) cells. (**A**) Timeline of the in vitro experiment. dHL-60 cells were pretreated with DOVO (0.8, 1.6, 3.2 μg/mL) for 6 h, followed by stimulation with 10 μg/mL LPS for 6 h; (**B**) Relative mRNA expression of IL-1β (*** *p* < 0.001, ** *p* < 0.01, N = 5); (**C**) Relative mRNA expression of IL-8 (**** *p* < 0.0001, *** *p* < 0.001, ** *p* < 0.01, N = 5); (**D**) Relative mRNA expression of ROS (** *p* < 0.01, N = 5); (**E**) Relative mRNA expression of CXCL2 (** *p* < 0.01, * *p* < 0.05, N = 5); (**F**) Immunofluorescence staining of CitH3 (red) and MPO (green) in dHL-60 cells (the red box is the magnified image, bar = 50 μm, the yellow arrows indicate the co-localization of MPO^+^ and CitH3^+^); (**G**) Western blot analysis of MPO and CitH3 protein expression, with GAPDH as the internal reference; (**H**) Quantitative analysis of CitH3^+^MPO^+^ fluorescence intensity (* *p* < 0.05, ** *p* < 0.01, N = 5). (CON, control; MOD, LPS-induced model; OL, low-dose DOVO; OM, medium-dose DOVO; OH, high-dose DOVO. Data are presented as mean ± SEM; ns, not significant).

Based on the successful establishment of the HL-60 cell differentiation model into neutrophil-like cells (dHL-60), an in vitro model of LPS-induced inflammation and neutrophil extracellular trap (NET) activation was further developed using dHL-60 cells. The cells were pretreated with *D. odorifera* volatile oil (DOVO) at concentrations of 0.8, 1.6, and 3.2 μg/mL, designated as the OL, OM, and OH groups, respectively, for 6 h, followed by stimulation with 10 μg/mL LPS for an additional 6 h (Figure 5A). qPCR analysis indicated that the relative mRNA levels of IL-1β, IL-8, ROS, and CXCL2 were markedly upregulated in the LPS-treated model group (MOD) compared to the normal control group (CON) (all * *p* < 0.001 or *p* < 0.01). In comparison to the MOD group, DOVO pretreatment differentially regulated the expression of these genes in a dose-specific manner. Specifically, IL-1β mRNA was significantly inhibited in the OL, OM, and OH groups (Figure 5B). IL-8 levels were significantly reduced only in the OL and OM groups, with no significant effect observed in the OH group (Figure 5C). For ROSs, only the OL group exhibited significant downregulation, while the OM and OH groups showed no significant differences (Figure 5D). Regarding CXCL2, a significant decrease was detected only in the OM group, while the OL and OH groups exhibited no statistically significant changes (Figure 5E). Western blot results (Figure 5G) demonstrated that the normalized relative expression levels of myeloperoxidase (MPO) and citrullinated histone H3 (CitH3), normalized to GAPDH, were significantly elevated in MOD cells (MPO: 1.00, CitH3: 1.00) compared to the CON group. Conversely, all DOVO dose groups exhibited a dose-dependent downregulation of the normalized expression of MPO and CitH3, with the OH group showing the most pronounced inhibitory effects (MPO: 0.31, CitH3: 0.60). Immunofluorescence co-localization results (Figure 5F,H) further corroborated these findings: only a minimal amount of CitH3^+^MPO^+^ double-positive signals was detected in the CON group, whereas the double-positive fluorescence intensity was significantly heightened in the MOD group, indicating substantial formation of NETs. In contrast, the CitH3^+^MPO^+^ double-positive fluorescence intensity in all DOVO dose groups diminished in a dose-dependent manner, with the fluorescence intensity in the OH group approaching that of the CON group. This suggests that DOVO effectively inhibits LPS-induced NET formation in dHL-60 cells. Collectively, these findings indicate that DOVO can mitigate LPS-induced neutrophil-related inflammatory injury in vitro by inhibiting the expression of inflammatory factors, reducing oxidative stress, and blocking the MPO-CitH3-mediated NET pathway.

## 3. Discussion

In the present study, the *D. odorifera* volatile oil (DOVO) exhibited a marked protective effect on rat myocardial microcirculatory disturbance induced by polyethylene microspheres and improved hemodynamic parameters. The major chemical constituents of DOVO were identified as three sesquiterpenoids, (3S,6R,7R)-3,7,11-trimethyl-3,6-epoxy-1,10-dodecadien-7-ol (22.4%), (3S,6S,7R)-3,7,11-trimethyl-3,6-epoxy-1,10-dodecadien-7-ol (15.3%), and *trans*-nerolidol (62.4%), together accounting for approximately 91% of the total peak area (Figure 1). Regarding the translational relevance and safety of DOVO doses, the doses used in this study were derived from clinical practice. According to the Chinese Pharmacopoeia, the recommended daily dose of *D. odorifera* crude drug for adults is 9–15 g per 60 kg body weight [28]. Based on body surface area conversion (rat/adult human conversion factor of 6.17), the equivalent rat crude drug dose is approximately 1 g/kg [29]. Using the experimentally determined extraction yield of 3.0065% (*v*/*w*) obtained from our batch of *D. odorifera* heartwood, the corresponding DOVO dose in rats was calculated as 30 mg/kg, which exactly matches our low dose (OL). The medium (60 mg/kg, OM) and high (120 mg/kg, OH) doses are 2-fold and 4-fold higher, respectively, designed to explore dose-dependent effects. DOVO has been reported to have very low oral toxicity at therapeutic doses. The injection of microspheres led to the obstruction of cardiac blood vessels, infiltration by inflammatory cells, increased neutrophil recruitment, elevated serum levels of IL-1β, TNF-α and IL-6, and enhanced formation of neutrophil extracellular traps (NETs), as indicated by an increase in CitH3^+^MPO^+^ neutrophils, PAD4/MPO expression, and serum reactive oxygen species (ROSs). In a dose-dependent manner, pretreatment with DOVO mitigated hemodynamic dysfunction, inflammatory response, and oxidative stress while inhibiting NET formation via the PAD4-MPO pathway. To further investigate the effects of DOVO on cardiac coronary microcirculatory inflammatory injury caused by NETs from neutrophils, an in vitro DOVO dHL-60 cell model was established. DOVO has been demonstrated to inhibit the mRNA levels of IL-1β, IL-8, CXCL2, and ROS. In addition, it also inhibits the protein levels of MPO/CitH3 in LPS-stimulated dHL-60 cells. According to the findings, DOVO can reduce microcirculation inflammatory injury by inhibiting oxidative stress, neutrophil recruitment, and PAD4-MPO-mediated NET formation. The development of cardiovascular and cerebrovascular disorders (CCDs) is characterized by microcirculatory disturbance [30], which is closely related to persistent inflammation [31,32], oxidation stress [33,34,35], and abnormal activation of neutrophils [36,37]. The microcirculation-improving traditional Chinese medicines displayed blood-activating and stasis-resolving properties, mainly through the regulation of vasodilation, increased blood flow and reduced inflammatory responses [38]. Nonetheless, the role of these drugs in neutrophil extracellular trap (NET) formation during microcirculatory injury is largely unknown. The medicinal plant *D. odorifera* is commonly used in clinics to enhance blood circulation and relieve pain [18]. Anti-inflammatory and antioxidant activities are properties shown to appear in its volatile oil (DOVO) [18]. Nevertheless, the microcirculatory protective effects of DOVO have not been associated with the regulation of neutrophil extracellular traps (NETs), which represents a research gap. This research study aims to provide evidence for DOVO alleviation of microcirculatory injury by targeting the redox-PAD4-MPO-NETs axis. This study showed that microsphere-induced microcirculatory disturbance causes persistent neutrophil recruitment and excessive NET formation in the heart and brain. In contrast, previous studies either focused on hemodynamic parameters or studied general inflammatory markers. Significantly, DOVO-mediated NETs inhibitory effects were validated in animals and in LPS-stimulated differentiated HL-60 cells, followed by reduced inflammation and oxidation responses. As far as we know, this is the first study showing that DOVO could enhance microcirculatory function by inhibiting PAD4-MPO mediated NET formation, therefore bridging the mechanistic link between the blood activation effect of DOVO and regulation of neutrophil-dependent inflammatory injury.

Several limitations of the present study warrant consideration. First, the protective effects of DOVO were evaluated solely in an acute microcirculatory disturbance model induced by polyethylene microspheres, which may not fully replicate the chronic and complex pathological processes associated with clinical cardiovascular and cerebrovascular diseases. Second, although the PAD4-MPO pathway has been identified as a significant target of DOVO, specific pathway intervention assays—such as genetic overexpression or pharmacological inhibition of PAD4—were not conducted to confirm a direct causal relationship. Third, while the major sesquiterpenoid components of DOVO were identified by HPLC, the specific compound(s) responsible for inhibiting NETs and enhancing microcirculation remain to be determined through further bioactivity-guided isolation. We acknowledge that GC/MS is the standard technique for volatile oil analysis. In our preliminary experiments, GC analysis was performed; however, the three major sesquiterpenoids could not be adequately separated on our GC column. Therefore, we switched to HPLC with a C18 column, which successfully resolved these components. Fourth, a notable discrepancy in experimental design was observed between the animal and cell models regarding the use of positive controls. Specifically, nicorandil and borneol were utilized as positive drugs in the animal model, whereas no positive controls were incorporated in the in vitro cell experiments. This design choice stemmed from the distinct pharmacological roles of these two agents and the research focus of each model: nicorandil is a clinically established therapeutic for cardiovascular disorders, whereas borneol is a traditional Chinese medicine known for its efficacy in promoting blood circulation, resolving stasis, and inducing resuscitation—qualities that render them suitable for comparative analysis in animal models of microcirculatory disturbance. However, in the in vitro setting, where the focus was on the direct inhibitory effect of DOVO on NET formation in neutrophils (the key inflammatory cells), no direct experimental evidence has been reported to substantiate that either nicorandil or borneol can specifically suppress NETs generation in neutrophils. Regarding the use of ATRA-differentiated HL-60 cells (dHL-60), we acknowledge that this cell line does not fully recapitulate all characteristics of primary human neutrophils. However, for the specific endpoints of this study—LPS-induced NET formation and the expression of key NET-related proteins (PAD4, MPO, CitH3)—dHL-60 cells have been validated as a practical and relevant model [39]. RNA-seq analysis has shown that differentiation increases the expression of NET-related genes [40]. Given the short lifespan and donor variability of primary neutrophils, dHL-60 cells remain a useful tool for mechanistic studies of NETosis, including our investigation of DOVO’s inhibitory effects.

Despite these limitations, our findings offer novel mechanistic insights into the microcirculatory protective effects of DOVO. Future studies will focus on screening and identifying the active constituents of DOVO that target NET formation, validating their efficacy in clinically relevant chronic disease models, and conducting specific pathway intervention experiments to further elucidate the mechanism by which DOVO regulates the oxidative stress-PAD4-MPO-NETs axis. Collectively, these findings suggest that DOVO may have beneficial effects on microcirculatory disturbance-related cardiovascular and cerebrovascular injury, warranting further investigation in chronic disease models and clinical settings. This study provides preliminary experimental evidence for the potential application of DOVO in improving microcirculatory function [41].

## 4. Materials and Methods

### 4.1. Plant Material

The heartwood of *D. odorifera* T. Chen was collected from Baisha Li Autonomous County, Hainan Province, China, and purchased from Bozhou Xindutang Pharmaceutical Co., Ltd. (Bozhou, China). The plant material was authenticated by Prof. Xiaomei Fu, School of Pharmacy, Jiangxi University of Chinese Medicine. A voucher specimen (No. [to be deposited]) has been deposited at the Herbarium of Jiangxi University of Chinese Medicine. The volatile oil was extracted by continuous heat reflux extraction and the extraction yield was determined to be 3.0065% (*v*/*w*, based on the dry weight of the heartwood). The oil was stored at 4 °C until use.

### 4.2. HPLC Analysis Method of D. odorifera Volatile Oil

The HPLC analysis of *D. odorifera* volatile oil (DOVO) was performed as follows. An appropriate amount of DOVO was mixed with methanol by vortexing to prepare a test solution at a concentration of 5 mg / mL, and 1.5 mL of this solution was transferred into an HPLC vial for subsequent analysis. The chromatographic separation was carried out on an Agilent ZORBAX SB-C18 column (4.6 × 250 mm, 5 μm) (Agilent Technologies, Santa Clara, CA, USA) using a mobile phase consisting of acetonitrile and 0.1% formic acid in water with a gradient elution program: acetonitrile was linearly increased from 60% to 90% (with a corresponding decrease of 0.1% formic acid in water from 40% to 10%) within 0–30 min. The detection wavelength was set at 210 nm, the column temperature was maintained at 25–30 °C, and the flow rate was 1.0 mL/min. Compound identification was achieved by comparing the retention times and UV spectral characteristics of each peak with those of reference standards.

### 4.3. Animals and Grouping

SPF-grade male SD rats were utilized in this study. Following a 5-day adaptive feeding period, the rats were randomly assigned to the following groups: Control group (CON), model group (Model), nicorandil group (NIC, 1 mg·kg^−1^), borneol group (BP, 200 mg·kg^−1^), low-dose DOVO (OL, 30 mg·kg^−1^), medium-dose group (OM, 60 mg·kg^−1^), and high-dose group (OH, 120 mg·kg^−1^). In compliance with the 3R principles (Replacement, Reduction, Refinement), all surgical procedures were performed under adequate anesthesia (urethane and isoflurane) to minimize animal suffering, and the number of animals used was statistically justified to achieve reliable results with the minimum required. The Control and Model groups were administered distilled water intragastrically, while the other groups received the corresponding drugs once daily for 7 consecutive days. All drugs were prepared as a water suspension immediately before use and administered intragastrically at a constant volume of 10 mL/kg body weight once daily for 7 consecutive days.

### 4.4. Establishment of Microsphere-Induced Microcirculatory Disturbance Model

On the sixth day of administration, 45 min after the last gavage, rats in the MOD, NIC, BP, OL, OM, and OH groups were injected with polyethylene microspheres (PM, 42 μm in diameter, 60,000 microspheres per rat) into the heart to induce acute microcirculatory disturbance. In contrast, rats in the CON group received an equivalent volume of saline. Drug administration continued for an additional day. At the conclusion of the experiment, blood and tissues from the heart, liver, spleen, lungs, kidneys, and brain were collected for subsequent analysis.

### 4.5. Hemodynamic Measurement

Subsequent to the final administration and modeling process, hemodynamic parameters including heart rate (HR), systolic blood pressure (SBP), diastolic blood pressure (DBP), and mean arterial pressure (MAP) were recorded utilizing a dedicated hemodynamic analysis system.

### 4.6. Pathomorphological Observation

Heart, liver, lung, kidney, and brain tissues were fixed, dehydrated, embedded, and sectioned. Hematoxylin–eosin (HE) staining was performed to observe vascular obstruction, tissue damage, and inflammatory cell infiltration.

### 4.7. Neutrophil Counting

Bone marrow and peripheral blood were collected, and the total number of neutrophils was counted using a F3AL250V automatic blood cell analyzer (Chongqing Nanfang Numerical Control Equipment Co., Ltd., Chongqing, China).

### 4.8. Measurement of Inflammatory Factors and Oxidative Stress

Serum levels of interleukin-1β (IL-1β, MM-0047R1), tumor necrosis factor-α (TNF-α, MM-0180R1), and reactive oxygen species (ROSs, MM-21264R1) were detected using commercial ELISA kits (Jiangsu Meimian Industrial Co., Ltd., Yancheng, China) according to the manufacturer’s instructions.

### 4.9. Flow Cytometric Analysis of NET Formation

Peripheral blood was collected to prepare single-cell suspensions. After incubation with fluorescently labeled antibodies against CitH3 and MPO, the proportion of CitH3^+^MPO^+^ double-positive neutrophils was analyzed by flow cytometry to evaluate NET formation.

### 4.10. Western Blot Analysis

Total protein was extracted from heart and brain tissues and dHL-60 cells. Protein expression levels of TNF-α, IL-6, PAD4, MPO, and CitH3 were detected by Western blot. Gray values were analyzed using ImageJ 1.53q software and normalized to GAPDH.

### 4.11. Cell Culture and Differentiation of HL-60 Cells

Human HL-60 cells were cultured in IMDM supplemented with 20% fetal bovine serum. To induce neutrophil-like differentiation, HL-60 cells were treated with 2 μM all-trans-retinoic acid (ATRA) for 7 days. The differentiation efficiency was verified by Wright–Giemsa staining and flow cytometry (CD33 and CD66b). The HL-60 cell line was obtained from the Cell Bank, Chinese Academy of Sciences, Shanghai, China.

### 4.12. LPS-Induced Inflammation and NET Formation in dHL-60 Cells

Differentiated HL-60 (dHL-60) cells were pretreated with 0.8, 1.6, and 3.2 μg/mL DOVO for 6 h, followed by stimulation with 10 μg/mL LPS for 6 h to establish an inflammatory injury and NET activation model in vitro. DOVO was added to the dHL-60 cell culture medium 6 h prior to LPS stimulation. The same volume of DMSO (final concentration < 0.1%) was added to the control and model groups to exclude solvent interference.

### 4.13. Quantitative Real-Time PCR (qPCR)

Total RNA was extracted from cells, and cDNA was synthesized by reverse transcription. The mRNA expression levels of IL-1β, IL-8, CXCL2, and ROS-related genes were detected by qPCR.

### 4.14. Immunofluorescence Staining

dHL-60 cells were fixed, permeabilized, and incubated with primary antibodies against MPO and CitH3, followed by fluorescent secondary antibodies. Nuclei were stained with DAPI. The formation of NETs was observed and analyzed using a SP8 laser scanning confocal microscope (Leica Microsystems, Wetzlar, Germany).

### 4.15. Statistical Analysis

All data were expressed as mean ± standard deviation (SD). One-way analysis of variance (ANOVA) followed by post hoc tests was used for multiple comparisons. A value of *p* < 0.05 was considered statistically significant.

## 5. Conclusions

In conclusion, the findings suggest that DOVO alleviates myocardial microcirculatory dysfunction and hemodynamic disorders in a rat model. Mechanistically, DOVO suppressed neutrophil recruitment, inflammation, and the PAD4-MPO pathway, thereby reducing NET formation. These effects were confirmed in LPS-stimulated neutrophil-like cells. These findings suggest that DOVO may exert cardioprotective effects by modulating neutrophil activation and NETs, though further studies are required to validate its therapeutic potential.

## Data Availability

The original contributions presented in this study are included in the article. Further inquiries can be directed to the corresponding authors. During the preparation of this manuscript, the authors used Deepseek for language polishing, grammar checking, and improving readability. These tools were used solely for linguistic refinement and not for generating, analyzing, or interpreting scientific content. The authors take full responsibility for the intellectual integrity and scientific accuracy of the work.
